# Supplementary material for: Cleavage of MALAT1 RNA by 14-nt sgRNA-guided tRNase ZL
Source: PLoS One. 2025 Sep 18;20(9):e0318968. doi: 10.1371/journal.pone.0318968 (PMC12445523; doi:10.1371/journal.pone.0318968)
Supplement: S1 Fig — (A) Secondary structures of pre-tRNA and micro-pre-tRNA. (B) Secondary structures of sgRNA/target RNA complexes. Arrows and bars denote cleavage sites and base pairings, respectively. N, S and T represent A, U, G, or C. Sequences of S in sgRNAs form base-pairings with sequences of T in target RNAs. (PDF) [file pone.0318968.s001.pdf]

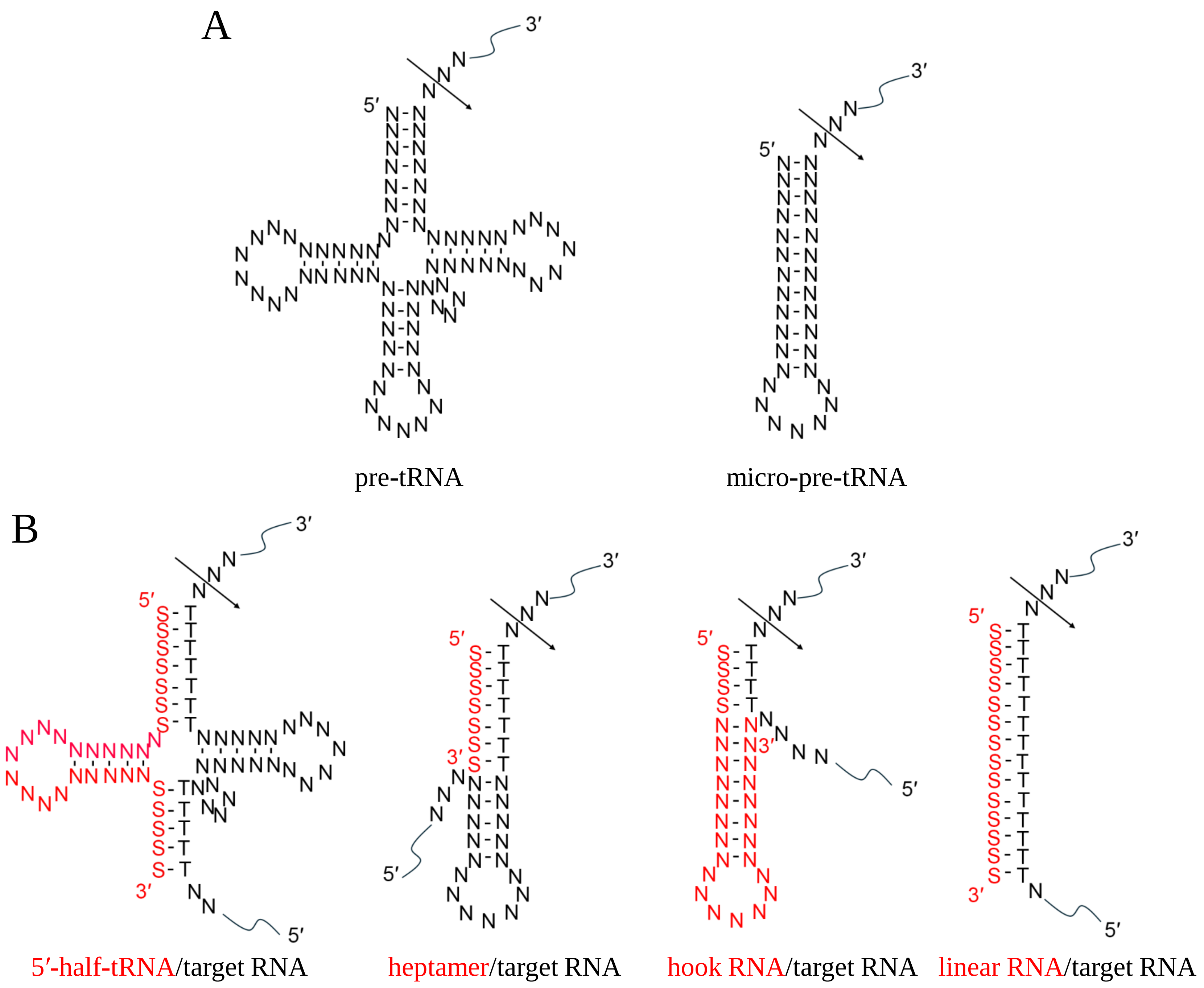
A
pre-tRNA
micro-pre-tRNA
B
5′-half-tRNA/target RNA
heptamer/target RNA
hook RNA/target RNA
linear RNA/target RNA

S1 Fig.
