# Supplementary material for: Cleavage of MALAT1 RNA by 14-nt sgRNA-guided tRNase ZL
Source: PLoS One. 2025 Sep 18;20(9):e0318968. doi: 10.1371/journal.pone.0318968 (PMC12445523; doi:10.1371/journal.pone.0318968)
Supplement: S2 Fig — GC content, melting temperature (Tm), and potential secondary structure of each sgRNA are shown. (PDF) [file pone.0318968.s002.pdf]

Name	Sequence	GC content (%)	Tm (°C)	Secondary structure
sgRM1	5′-UAGGAUUCUAGACA-3′	36	38	-
sgRM2	5′-UGGUUAUGACUCAG-3′	43	40	-
sgRM3	5′-AUUGCCUCUUCAUU-3′	36	38	-
sgRM4	5′-CCUUCUGCCUUAGU-3′	50	42	-
sgRM5	5′-CUUUUGCAUUUCCC-3′	43	40	-
sgRM6	5′-AAUCCCCUAGGGAA-3′	50	42	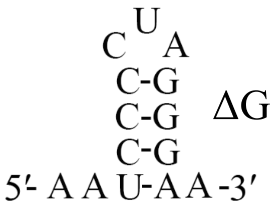 $\Delta G = -3.9$ kcal/mol
sgNC1	5′-CCCCCCCCCCCCCC-3′	100	56	-
sgNC2	5′-CUCUCUCUCUCUCU-3′	50	42	-

S2 Fig.
