# Supplementary figures and images for: Cleavage of MALAT1 RNA by 14-nt sgRNA-guided tRNase ZL
Source: PLoS One. 2025 Sep 18;20(9):e0318968. doi: 10.1371/journal.pone.0318968 (PMC12445523; doi:10.1371/journal.pone.0318968)

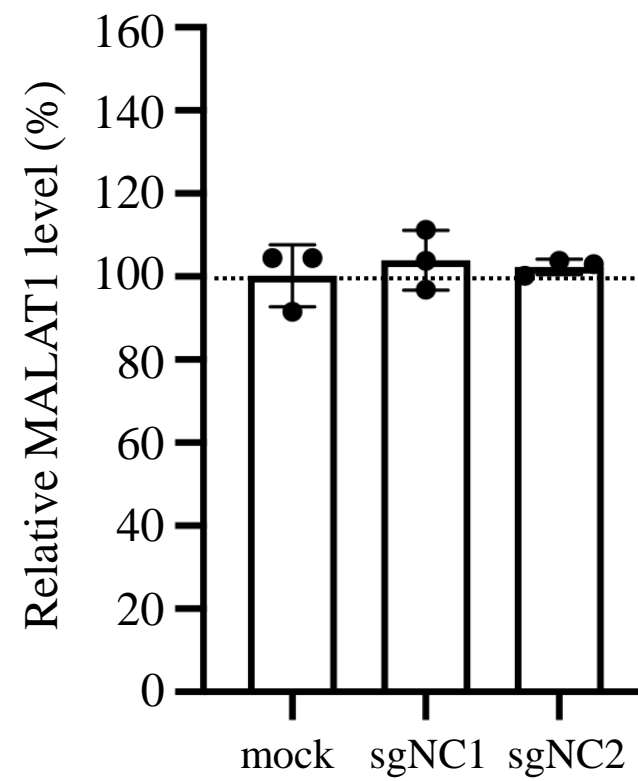

Supplement: S3 Fig — Human A549 cells were transfected without (mock) or with sgNC1 or sgNC2 (200 nM). After 96-hr culture, a MALAT1 RNA amount was measured by qRT-PCR, normalized against a β-actin mRNA amount, and expressed as a percentage relative to that of mock control cells. Values are mean ± SD for three biological replicates. (PDF) [file pone.0318968.s003.pdf]

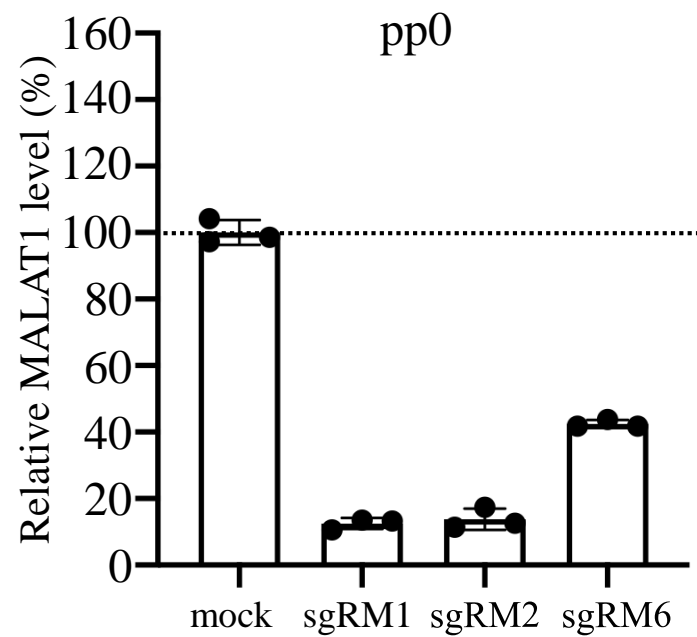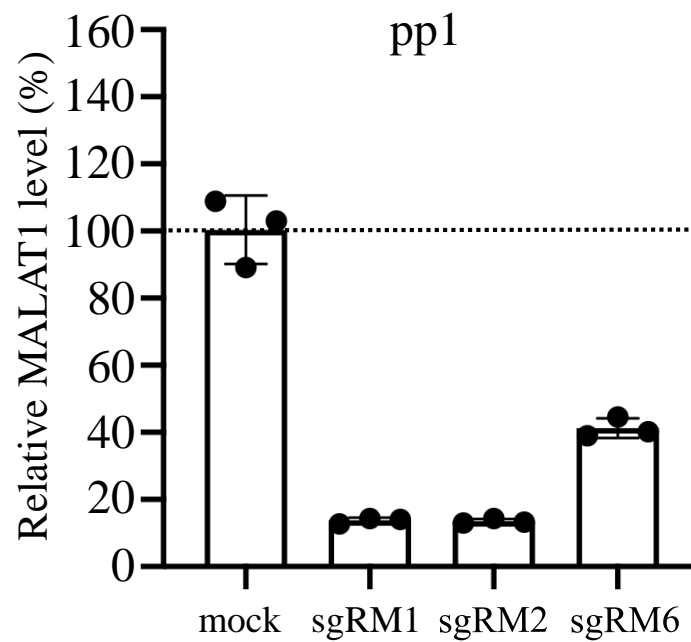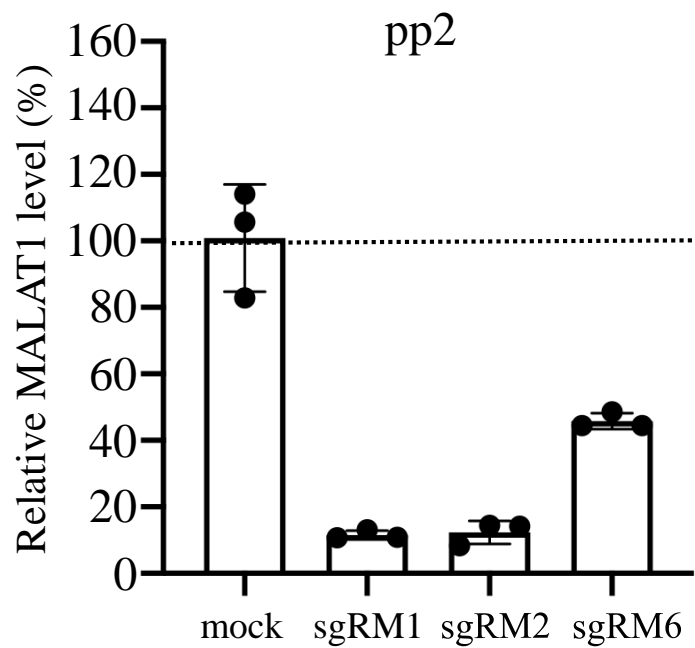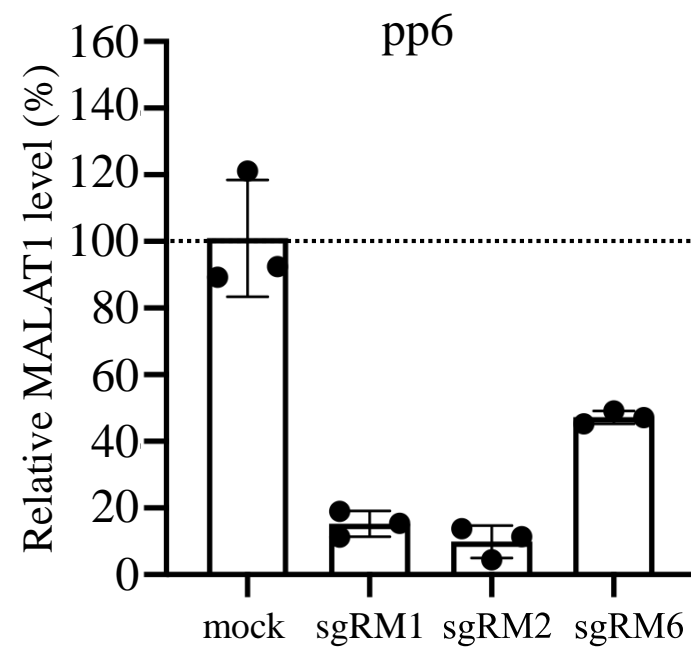

Supplement: S4 Fig — A549 cells were transfected without (mock) and with each of sgRM1, sgRM2 or sgRM6 (200 nM). After 96-hr culture, a MALAT1 RNA amount was measured by qRT-PCR with a primer pair, pp0, pp1, pp2 or pp6, normalized against a β-actin mRNA amount, and expressed as a percentage relative to that of mock control cells. Values are mean ± SD for three technical replicates. (PDF) [file pone.0318968.s004.pdf]

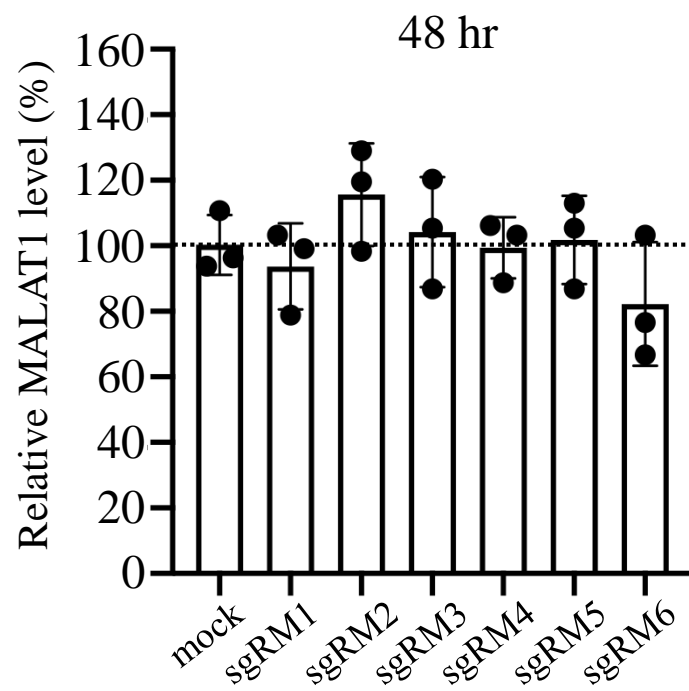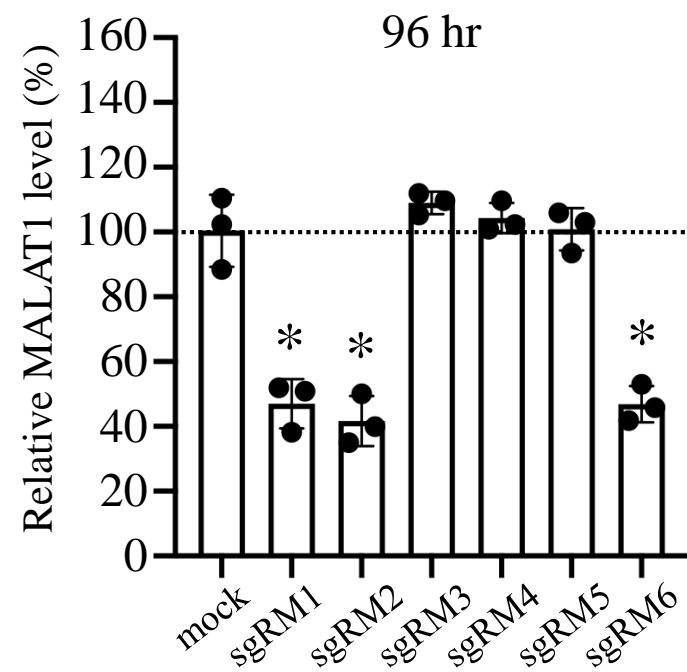

Supplement: S5 Fig — Human HEK293 cells were transfected without (mock) and with each of the sgRNA sgRM1 − sgRM6 (200 nM). After 48- and 96-hr culture, a MALAT1 RNA amount was measured by qRT-PCR with a primer pair, pp0, normalized against a β-actin mRNA amount, and expressed as a percentage relative to that of mock control cells. Values are mean ± SD for three biological replicates. Asterisk, p < 0.05. (PDF) [file pone.0318968.s005.pdf]

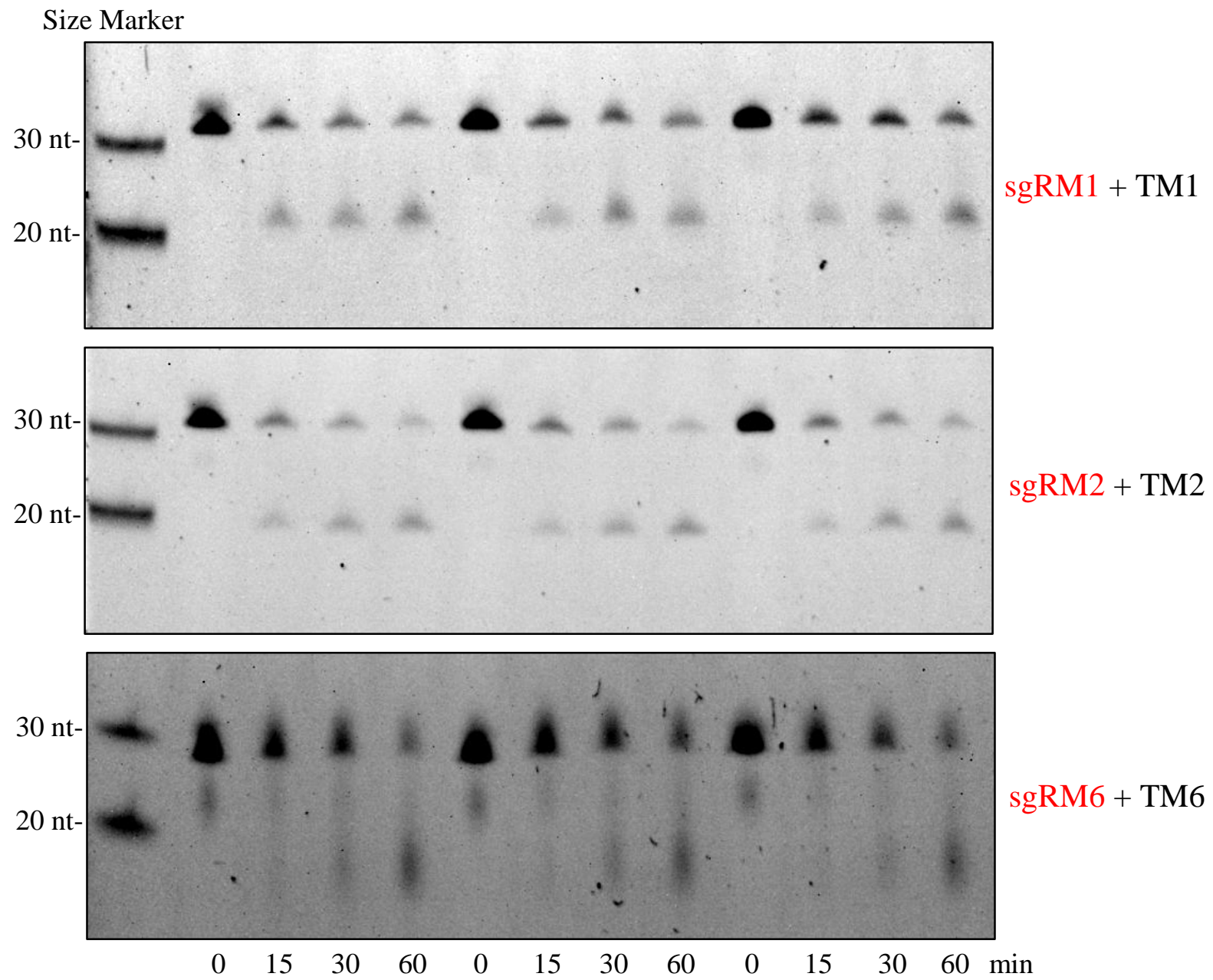

Supplement: S6 Fig — The 30-nt 5′-FAM-labeled MALAT1 fragments TM1, TM2 and TM6 were incubated in the presence of sgRM1, sgRM2 and sgRM6, respectively, with recombinant human tRNase ZL at 37°C for 0, 15, 30 and 60 min. A cleavage product was analyzed on a denaturing 12.5% polyacrylamide gel. Each assay was repeated three times. (PDF) [file pone.0318968.s006.pdf]

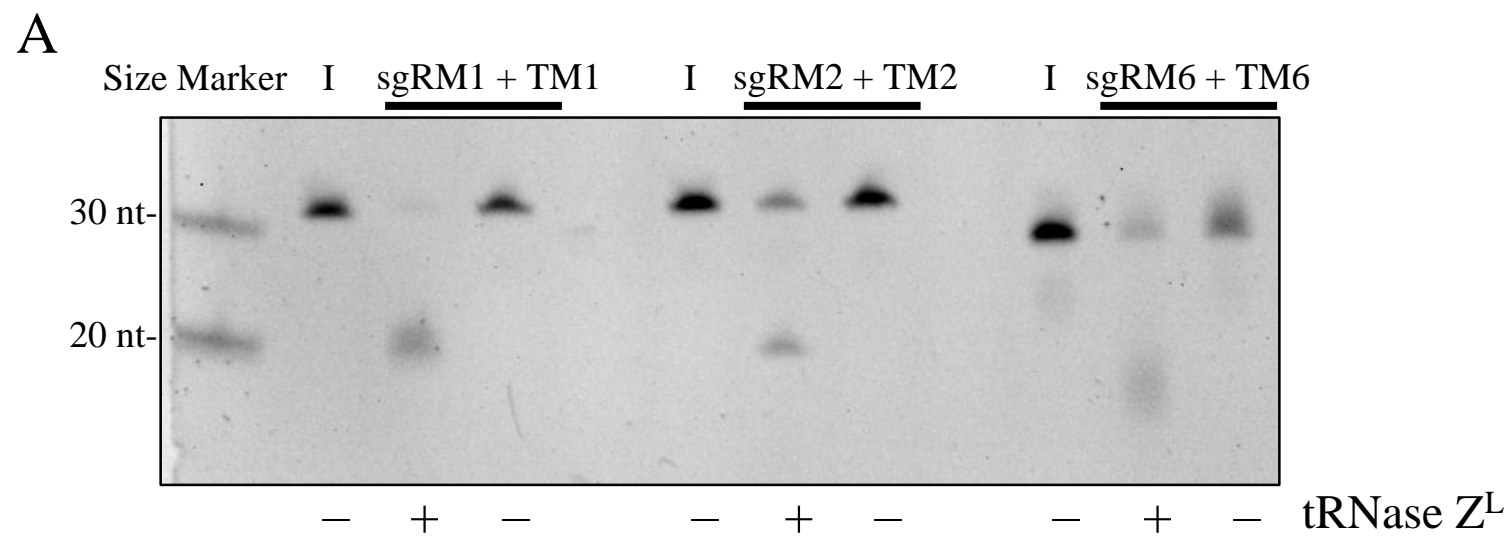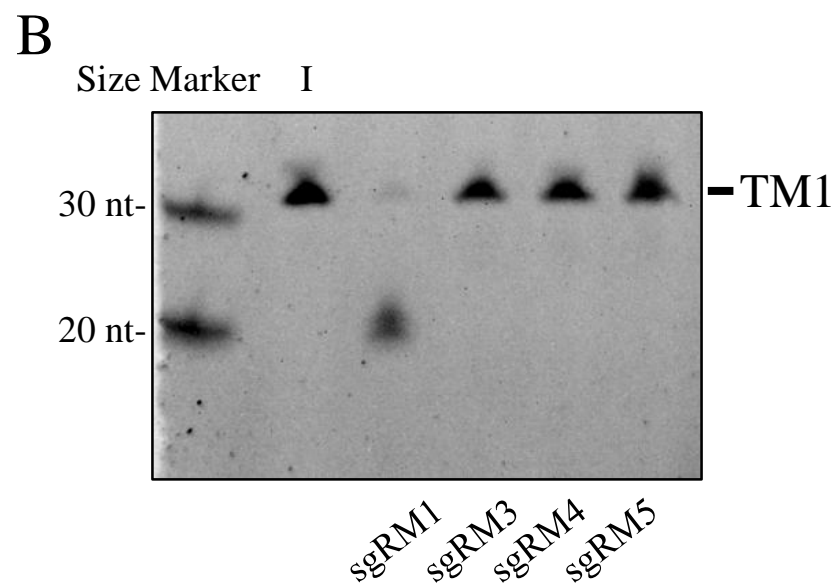

Supplement: S7 Fig — (A) The 5′-FAM-labeled MALAT1 fragments TM1, TM2 and TM6 were incubated in the presence of sgRM1, sgRM2 and sgRM6, respectively, with or without recombinant human tRNase ZL at 37°C for 60 min. (B) The 5′-FAM-labeled TM1 was incubated in the presence of sgRM1, sgRM3, sgRM4 or sgRM5 with recombinant human tRNase ZL at 37°C for 60 min. A cleavage product was analyzed on a denaturing 12.5% polyacrylamide gel. I, input substrate RNA. (PDF) [file pone.0318968.s007.pdf]

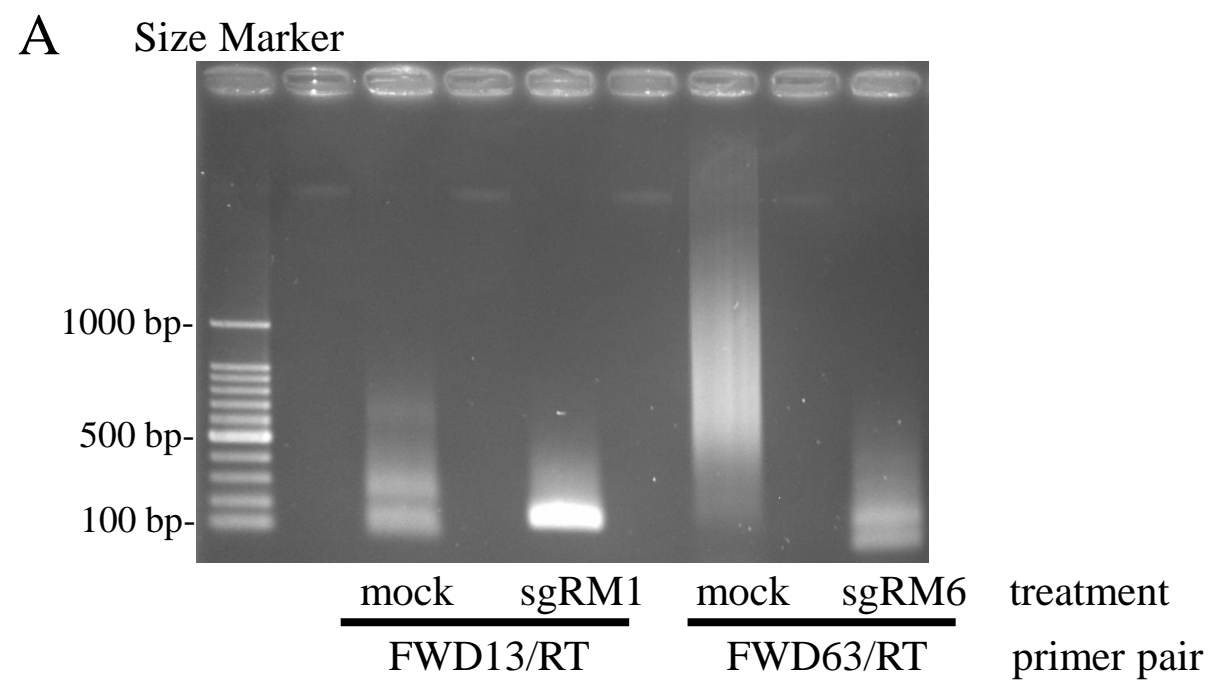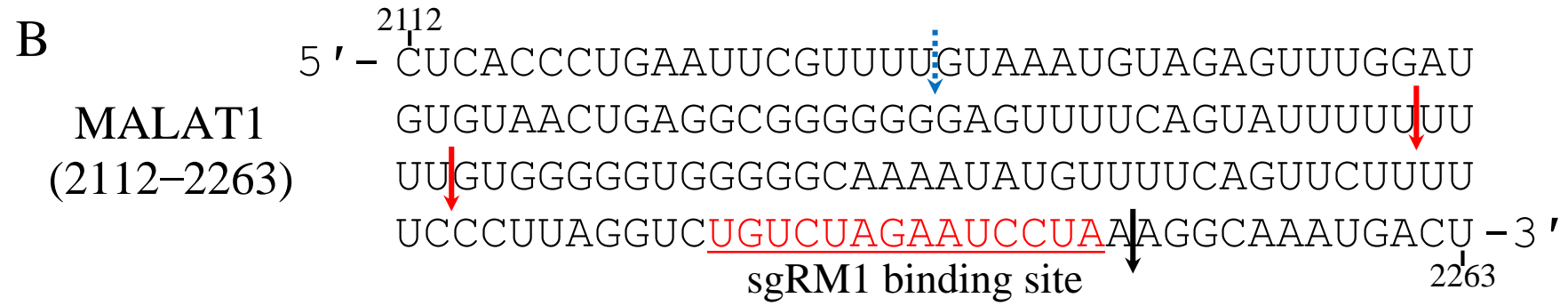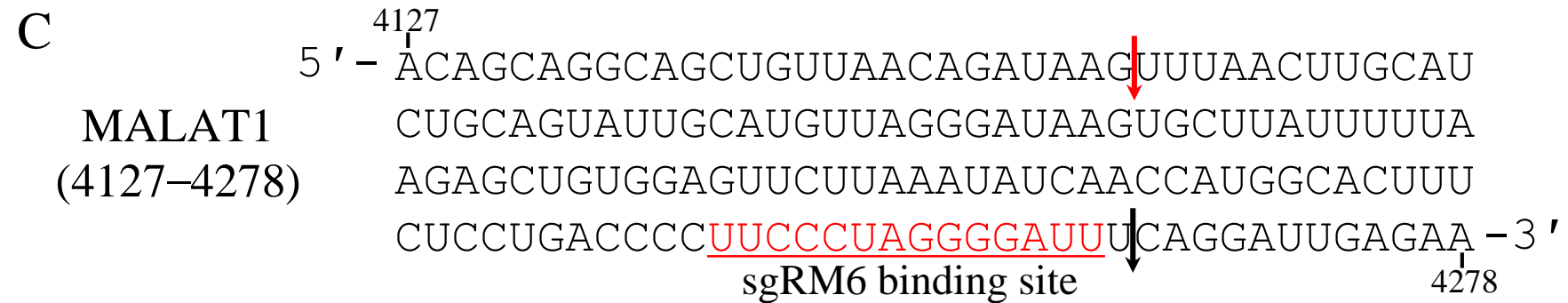

Supplement: S8 Fig — (A) PCR products obtained from the total RNA samples from mock-, sgRM1- and sgRM6-treated cells are shown on a 1.5% agarose gel. The nested primer pair FWD13/RT or FWD63/RT was used for 40-cycle PCR-amplification. (B) A partial MALAT1 sequence including the expected sgRM1-guided tRNase ZL cleavage site (denoted by a black arrow). (C) A partial MALAT1 sequence including the expected sgRM6-guided tRNase ZL cleavage site (denoted by a black arrow). Red arrow, the 3′-end of a 5′-cleavage product detected in sgRM1- or sgRM6-treated cells; broken arrow, the 3′-end of a 5′-cleavage product detected in mock-treated cells. (PDF) [file pone.0318968.s008.pdf]

Hoechst33342 [ $\lambda_{\text{ex}}$ : 352 nm]

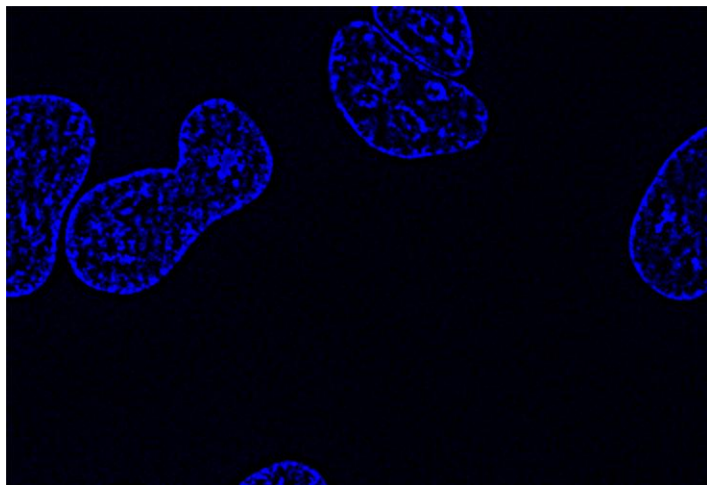

[ $\lambda_{\text{ex}}$ : 579 nm]

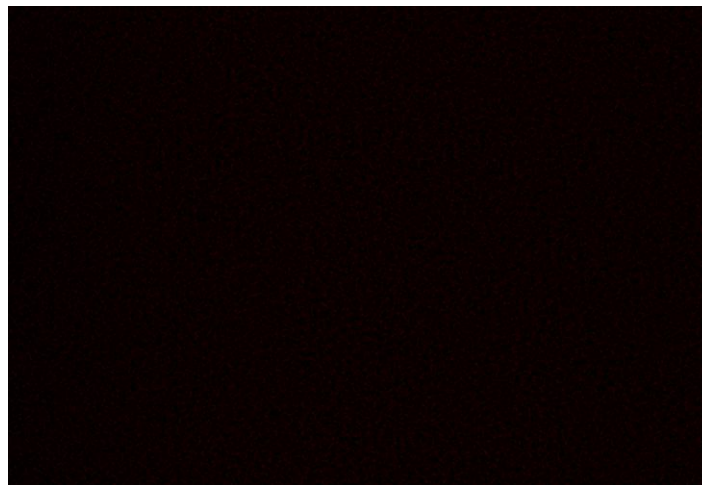

[ $\lambda_{\text{ex}}$ : 490 nm]

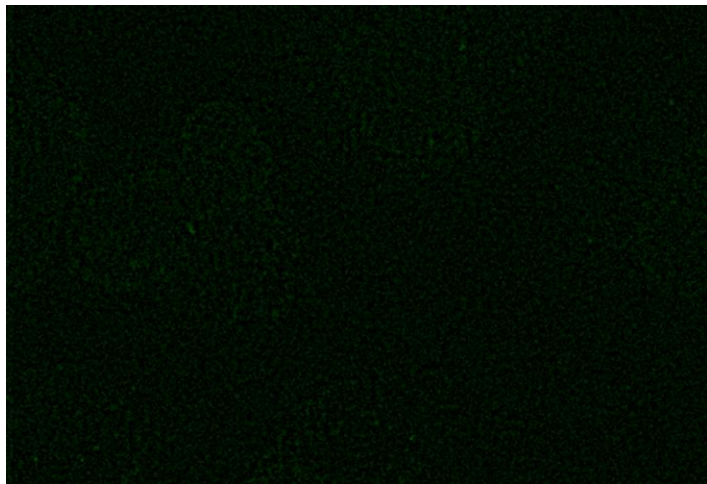

Merge

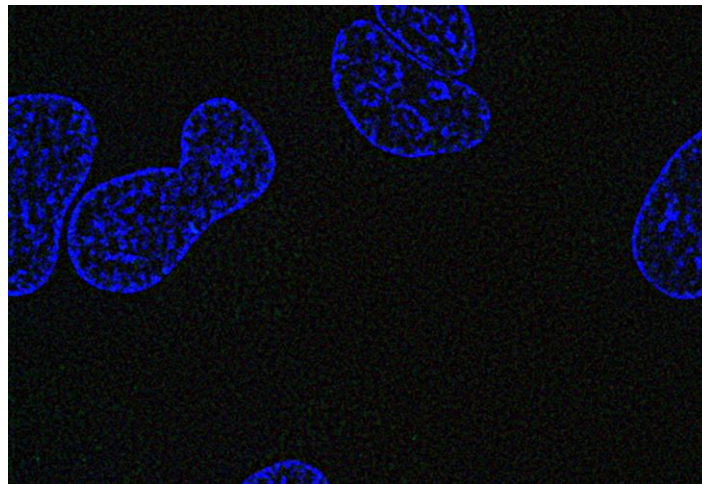

20  $\mu\text{m}$

Supplement: S9 Fig — As a control experiment, A549 cells were mock-transfected. After 48-hr culture, the cells were incubated with an Alexa488-conjugated secondary antibody without incubating with primary antibodies against a human tRNase ZL peptide, and analyzed with a fluorescence microscope. Hoechst 33342 was used to stain the nucleus. Each image was taken by irradiating a laser beam of the indicated wave length. (PDF) [file pone.0318968.s009.pdf]

A

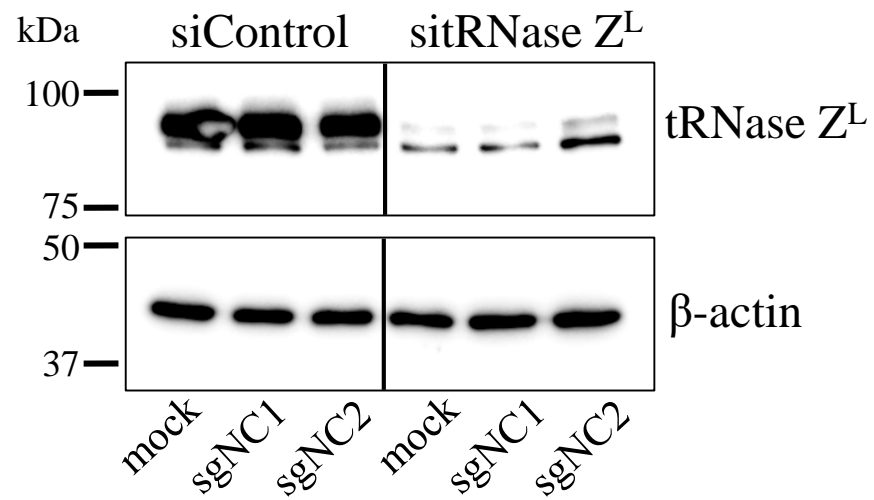

B

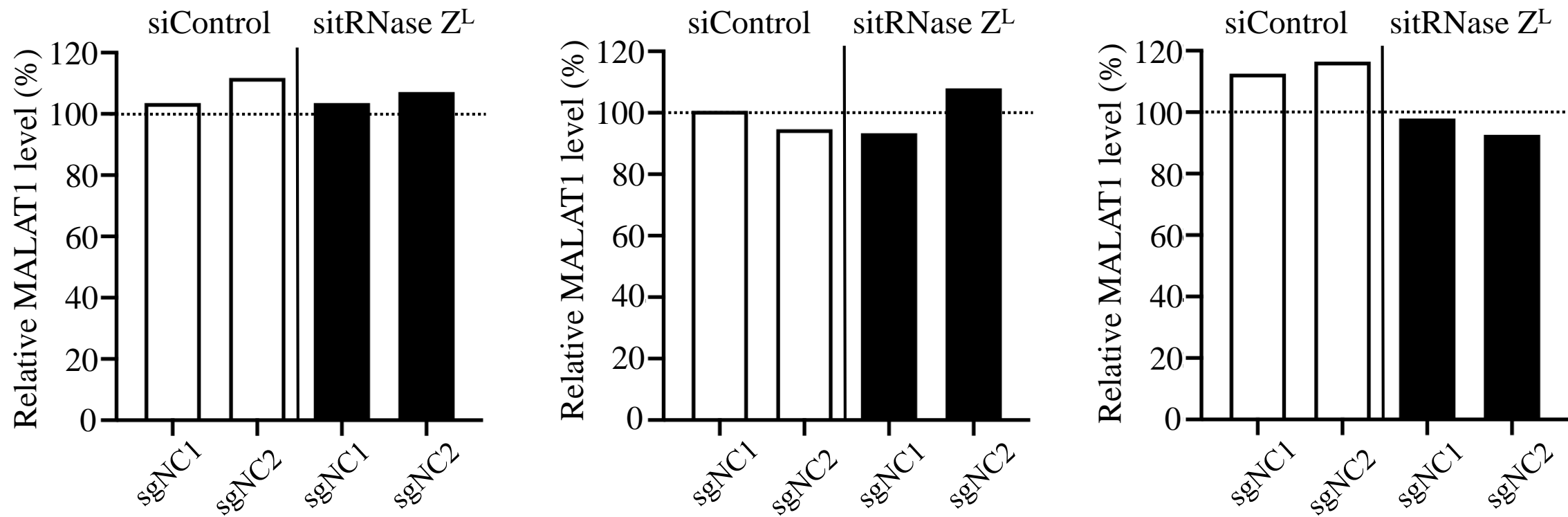

Supplement: S10 Fig — Twenty-four hr after A549 cells were transfected with siControl or sitRNase ZL, the cells were transfected without (mock) or with each of sgNC1 or sgNC2 (200 nM) and cultured further. After 96-hr culture, total cellular protein and RNA were prepared. (A) tRNase ZL and β-actin protein levels were analyzed by Western blotting. (B) A MALAT1 RNA amount was measured by qRT-PCR, normalized against a β-actin mRNA amount, and expressed as a percentage relative to that of mock control cells. Data were from three biological replicates. (PDF) [file pone.0318968.s010.pdf]

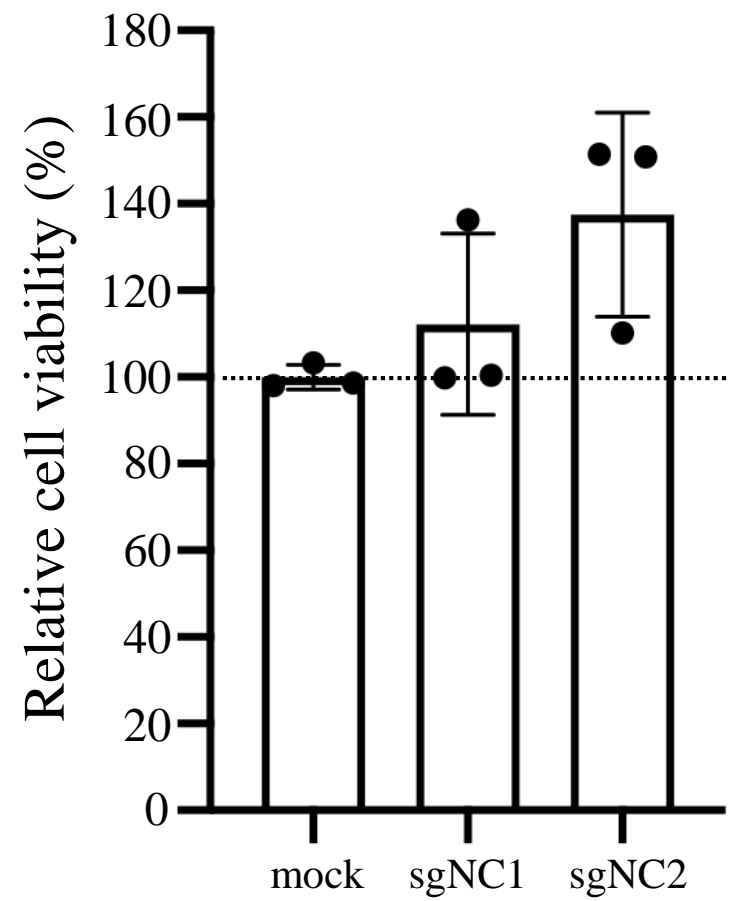

Supplement: S11 Fig — A549 cells were transfected without (mock) or with sgNC1 or sgNC2 (200 nM), and after 96-hr culture, cell viability was measured. The cell viability is expressed as a percentage relative to that of untreated cells. Values are mean ± SD for three biological replicates. (PDF) [file pone.0318968.s011.pdf]

A

B

C

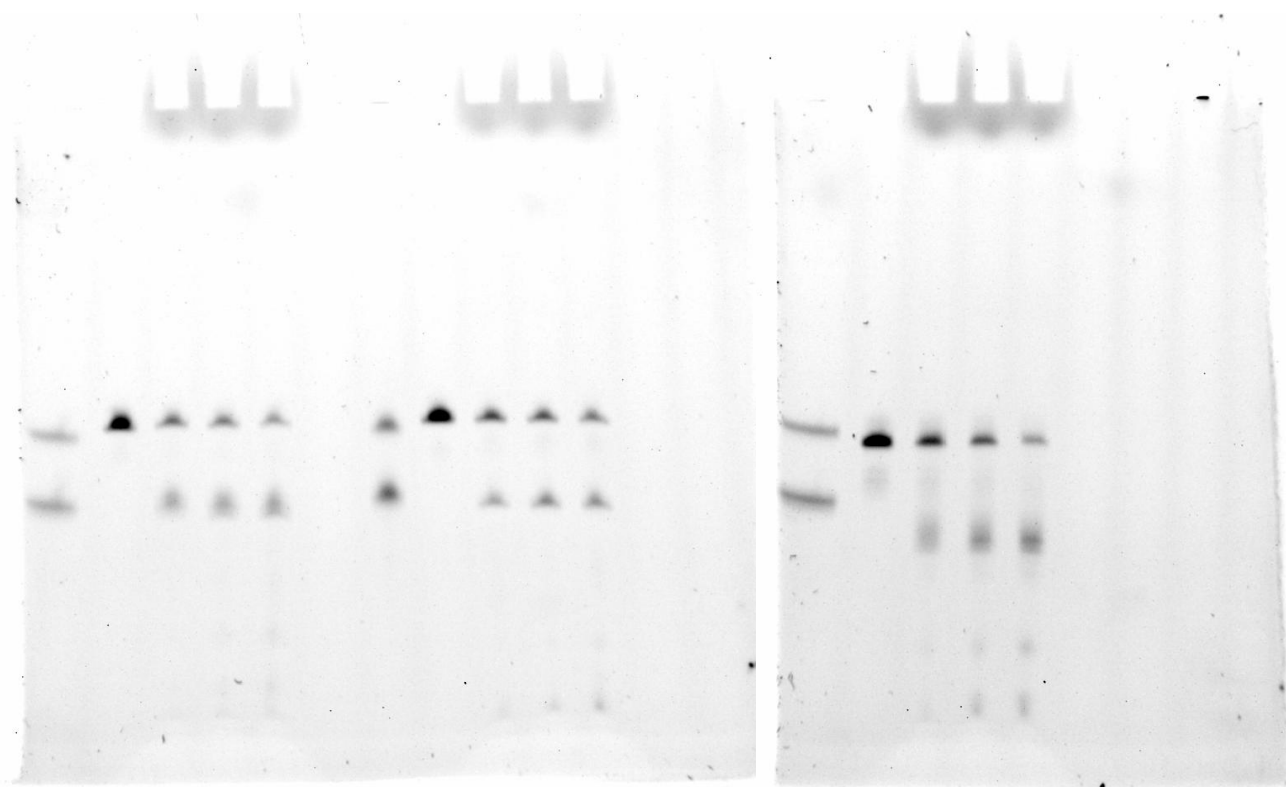

Fig. 3

tRNase Z<sup>L</sup>

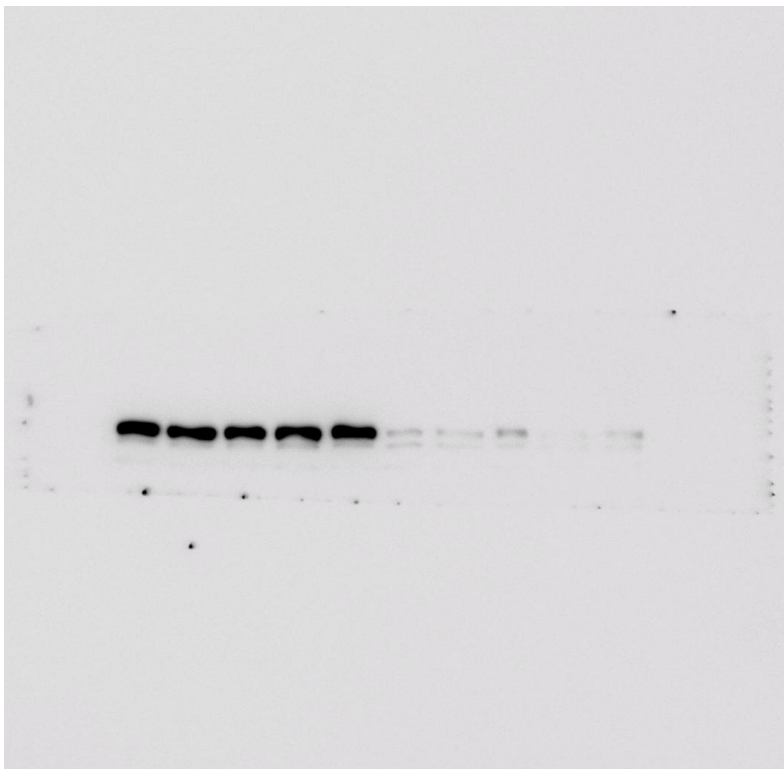

$\beta$ -actin

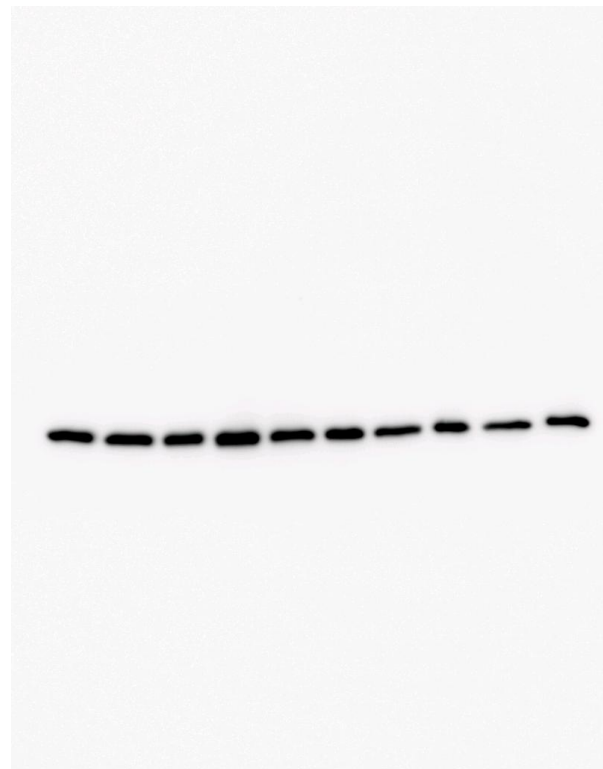

Fig. 5A

sgRM1 + TM1

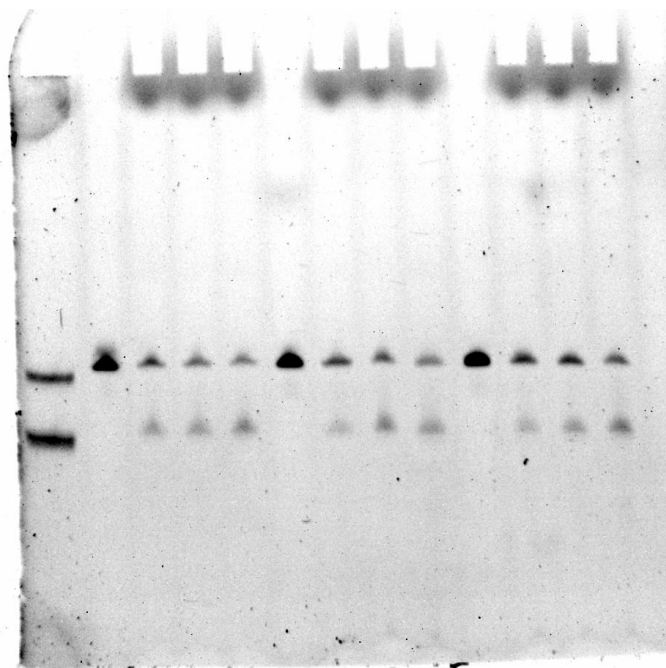

sgRM2 + TM2

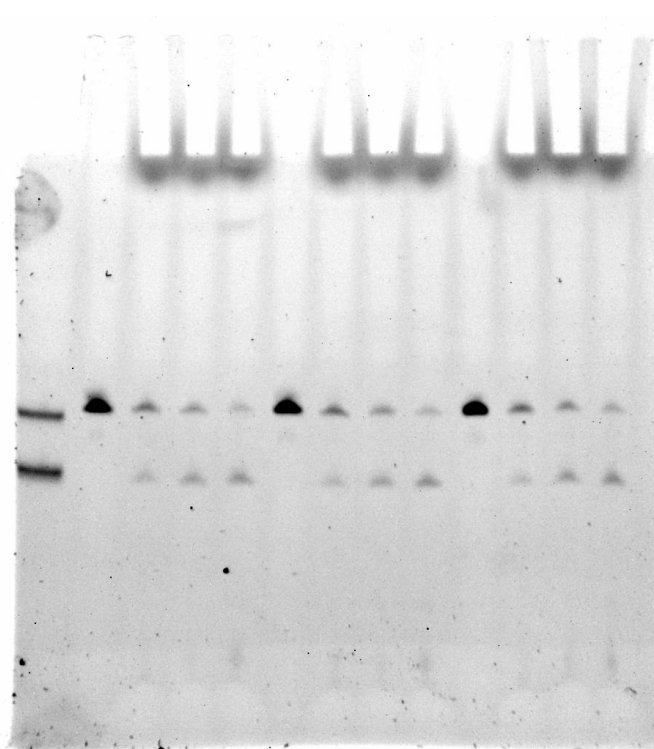

sgRM6 + TM6

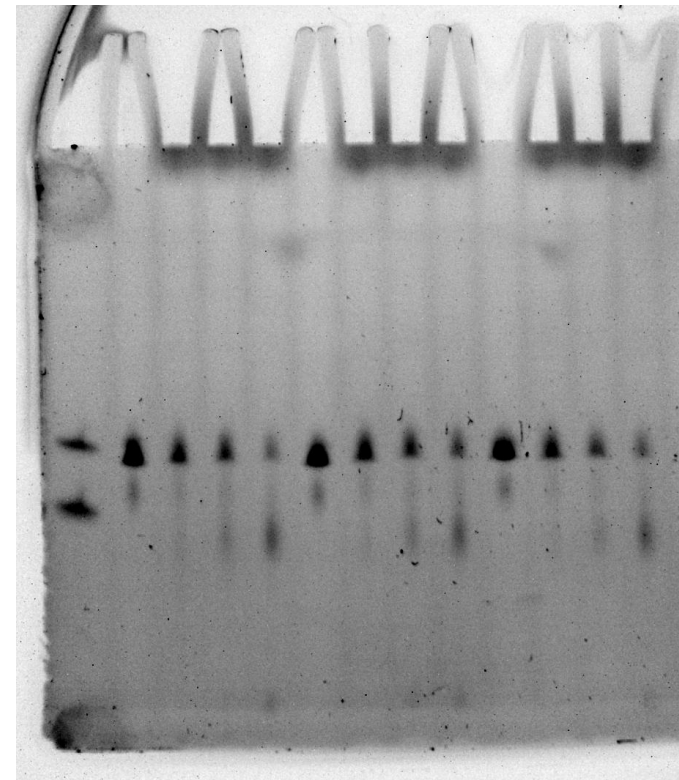

A

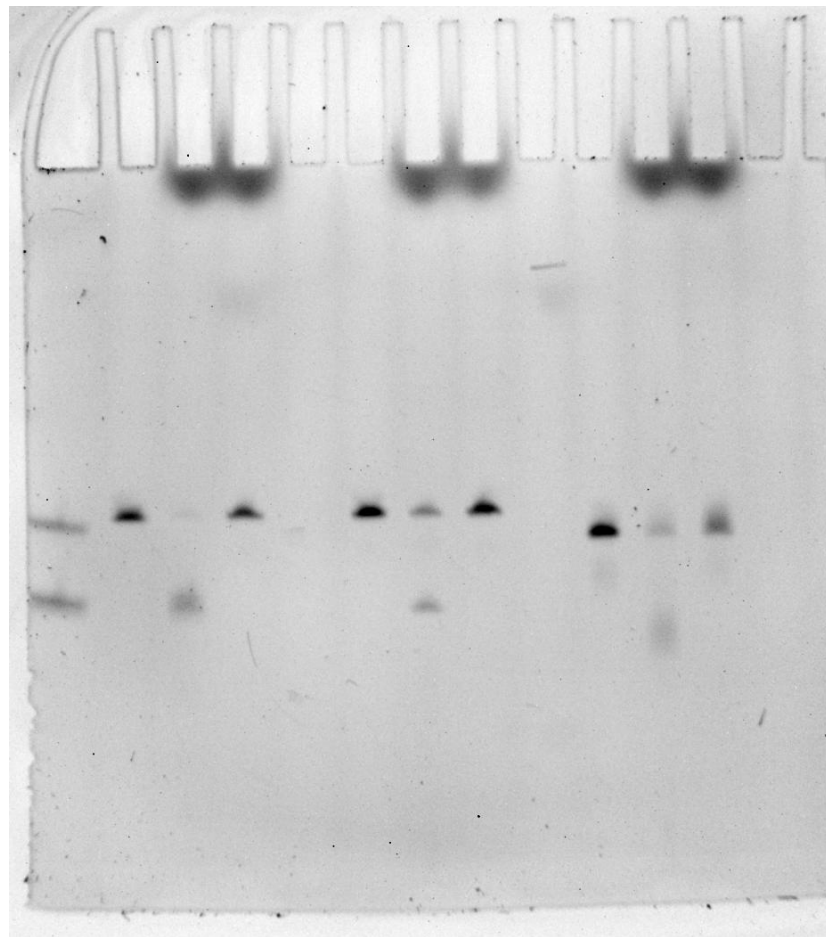

B

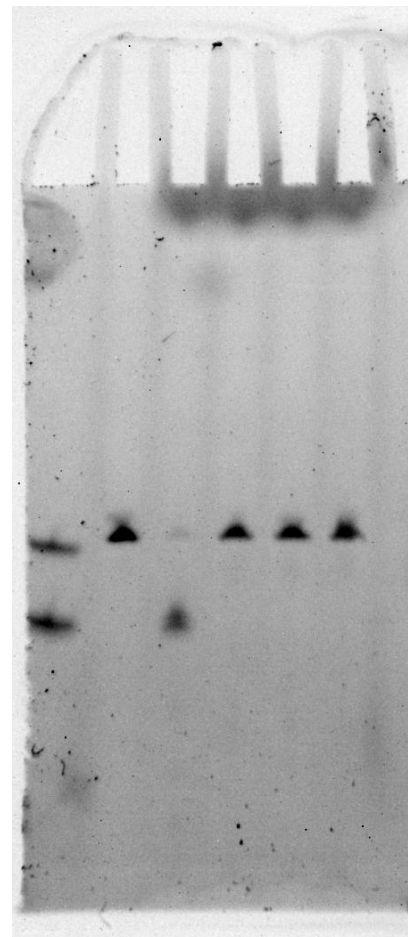

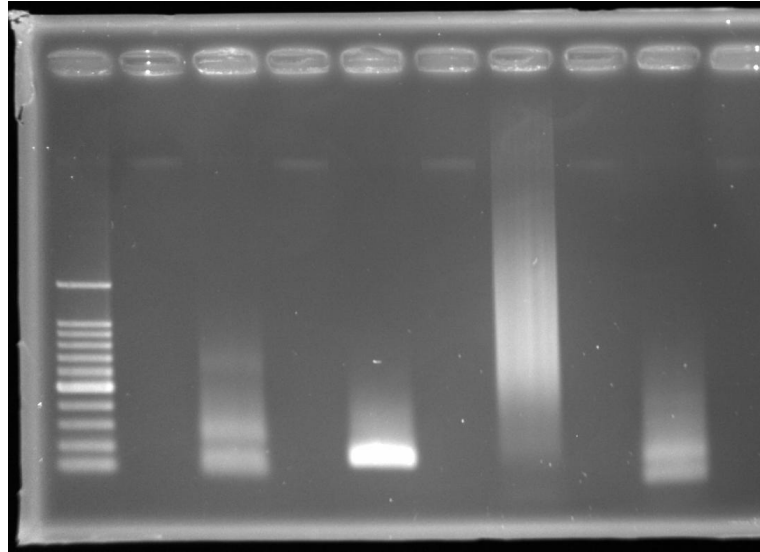

tRNase Z<sup>L</sup>

β-actin

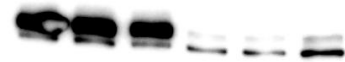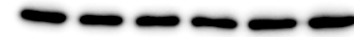

Supplement: S1_raw_images — (PDF) [file pone.0318968.s013.pdf]
