# Supplementary material for: Cleavage of MALAT1 RNA by 14-nt sgRNA-guided tRNase ZL
Source: PLoS One. 2025 Sep 18;20(9):e0318968. doi: 10.1371/journal.pone.0318968 (PMC12445523; doi:10.1371/journal.pone.0318968)
Supplement: Attachment — Submitted filename: Response to Reviewer (PLOS ONE)_R2.docx [file pone.0318968.s016.docx]

Response to Reviewer #4

The authors have addressed some but not all of the comments by this reviewer. However, the authors performed some experiments that can be accepted as substitutes for the requested approaches. This reviewer recommends publication after minor revision.

NEW COMMENTS:

SFig 9:

This Figure is called in the text as: “The sgRNA was observed to be distributed ubiquitously in A549 cells 48 hr after transfection, with higher density in the nucleus (Fig 4 and S9 Fig.)”. On the other hand, the Figure legend to SFig. 9 reads: “Fluorescence microscope image of mock-transfected A549 cells. As a control experiment, A549 cells were mock-transfected. After 48-hr culture, the cells were incubated with an Alexa488-conjugated secondary antibody without incubating with primary antibodies against a human tRNase ZL peptide, and analyzed with a fluorescence microscope. Hoechst 33342 was used to stain the nucleus. Each image was taken by irradiating a laser beam of the indicated wave length.”

>>To this reviewer there is some discrepancy between the two statements, especially since SFig. 9 does not show only the nuclear stain and no signal in the other two emission channels.

The control experiment shown in S9 Fig. has been performed to exclude the possibility of non-specific signals, which was pointed out by Reviewer #2. In this experiment, where the Alexa568-labeled sgRNA and the primary antibodies against a human tRNase Z^L^ peptide were not added, we observed no significant signal by irradiating laser beams of 579 and 490 nm. This observation would exclude the possibility of non-specific signals.
